# Using mutagenesis to explore conserved residues in the RNA-binding groove of influenza A virus nucleoprotein for antiviral drug development

**DOI:** 10.1038/srep21662

**Published:** 2016-02-26

**Authors:** Chia-Lin Liu, Hui-Chen Hung, Shou-Chen Lo, Ching-Hui Chiang, I-Jung Chen, John T.-A. Hsu, Ming-Hon Hou

**Affiliations:** 1 National Chung Hsing University, Department of Life Science, Taichung, 40227, Taiwan; 2 National Health Research Institutes, Institute of Biotechnology and Pharmaceutical Research, Miaoli, 35053, Taiwan; 3 National Chung Hsing University, Institute of Genomics and Bioinformatics, Taichung, 40227, Taiwan; 4 National Chung Hsing University, Institute of Biotechnology, Taichung, 40227, Taiwan

## Abstract

Nucleoprotein (NP) is the most abundant type of RNA-binding viral protein in influenza A virus–infected cells and is necessary for viral RNA transcription and replication. Recent studies demonstrated that influenza NP is a valid target for antiviral drug development. The surface of the groove, covered with numerous conserved residues between the head and body domains of influenza A NP, plays a crucial role in RNA binding. To explore the mechanism by which NP binds RNA, we performed a series of site-directed mutagenesis in the RNA-binding groove, followed by surface plasmon resonance (SPR), to characterize the interactions between RNA and NP. Furthermore, a role of Y148 in NP stability and NP-RNA binding was evaluated. The aromatic residue of Y148 was found to stack with a nucleotide base. By interrupting the stacking interaction between Y148 and an RNA base, we identified an influenza virus NP inhibitor, (E, E)-1,7-bis(4-hydroxy-3-methoxyphenyl) -1,6-heptadiene-3,5-dione; this inhibitor reduced the NP’s RNA-binding affinity and hindered viral replication. Our findings will be useful for the development of new drugs that disrupt the interaction between RNA and viral NP in the influenza virus.

Influenza is an infectious disease of birds and mammals caused by the influenza viruses belonging to the family Orthomyxoviridae^1^. The sudden swine-origin influenza virus H1N1v pandemic outbreak in 2009 caused 18,000 deaths^1^,^2^. The viral surface proteins hemagglutinin and neuraminidase have played important roles in antiviral drug discoveries and provide crucial neutralization against the virus^3^. Tamiflu (oseltamivir), which is a neuraminidase inhibitor, is used to treat flu infection^4^,^5^,^6^,^7^. However, several H1N1 influenza strains are resistant to Tamiflu because they contain the H274Y mutation in neuraminidase. Thus, new anti-influenza drugs are urgently needed.

Influenza A virus nucleoprotein (NP) is a major virion structural protein that has been predicted to interact with negative-strand viral RNA during viral nucleocapsid formation^8^. NP encapsulates the viral genome for RNA transcription, replication, virus packaging, and intracellular trafficking, and it also functions as a key adapter molecule between viral and host cell processes^9^. NP has been shown to cooperatively interact with RNA. In addition, NP interacts with a wide variety of viral and cellular macromolecules, including two subunits of the viral RNA-dependent RNA polymerase, viral matrix, actin, components of the nuclear import/export apparatus, and a nuclear RNA helicase^10^. According to protein sequence alignment, the 498-aa NP is highly conserved among influenza viruses. The multifunctional capabilities of NP in the viral life cycle makes this protein an attractive target for drug development^11^,^12^.

A substantial quantity of RNA is wrapped around each NP monomer, with a stoichiometric ratio of 20 nucleotides of RNA per 1 NP^13^. The NP crystal structure reveals that RNA molecules likely bind to a deep groove located between the head and body domains on the exterior of the NP oligomer^14^,^15^. Several residues that are critical for RNA binding and virus infectivity in the influenza A virus NP have been identified^16^,^17^. Ye *et al.* have been reported that the tail loop binding pocket as a potential target for antiviral development^14^. Le *et al.* reported that several NP mutations that affected the efficient incorporation of multiple viral-RNA (vRNA) segments into progeny virions even though a single vRNA segment was incorporated efficiently^16^. However, understanding structural and mechanistic information regarding influenza A virus NP and its interactions with RNA should facilitate the discovery of agents that specifically block the formation of ribonucleoprotein (RNP) during viral genome replication.

Accordingly, we proposed that the surface of the groove, which contains numerous conserved residues (including Y148, R150, R152, R156, R174, R175, K184, R195, R199, R213, R214, R221, R236) is able to interact with the RNA residue (Fig. 1). In this study, we performed a series of site-directed mutagenesis to explore the mechanism by which the NP binds RNA, followed by surface plasmon resonance (SPR) to monitor the binding between various mutants and RNA. Furthermore, a role of Y148 in the protein stability of NP and the binding of NP to RNA was evaluated. An aromatic residue, Y148 was also found to stack its benzene ring with a nucleotide base. By targeting Y148, we identified an influenza virus NP inhibitor, H7 [(E,E) -1,7-bis(4-hydroxy-3-methoxyphenyl)-1,6-heptadiene-3,5-dione], which reduced the NP’s RNA-binding affinity and hindered viral replication. Finally, we present a structural model of the influenza NP in complex with RNA, which clearly illustrates the critical role of Y148.

## Results

### Characterization of RNA-binding activity of wild-type and mutant nucleoproteins from H1N1

**Figure 1:**
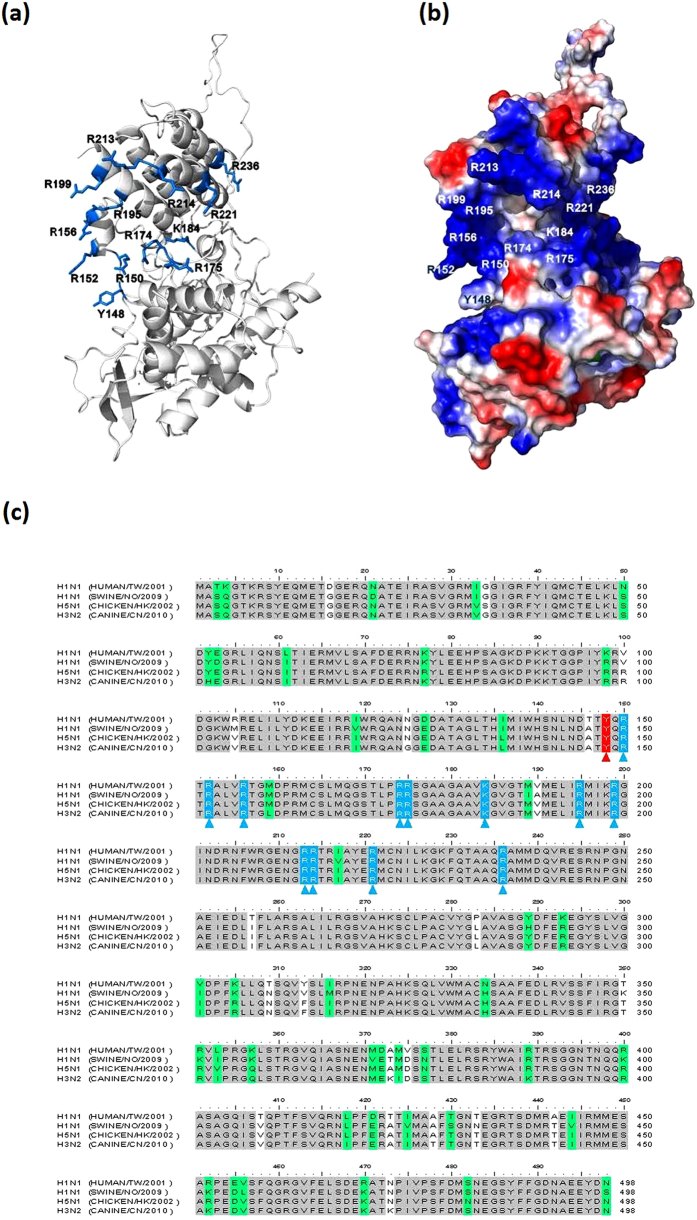
(**a**) Structural model of the influenza A virus (H1N1) nucleoprotein (NP). The 13 conserved residues that were substituted by alanine to analyze RNA-binding ability are marked. (**b**) Surface representation of the homology model of the influenza A virus (H1N1) NP: electrostatic potentials are blue (positive) or red (negative). (**c**) Amino acid pairwise sequence alignment of the NPs of the H1N1 strain (A/HUMAN/TW/2001), (A/SWINE/NO/2009), H5N1 (A/CHICKEN/HK/2002), and H3N2 (CANINE/CN/2010). Tyrosine (Y), arginine (R), and lysine (K) that were substituted by alanine are highlighted with red and blue, respectively, and indicated with triangles.

**Figure 2:**
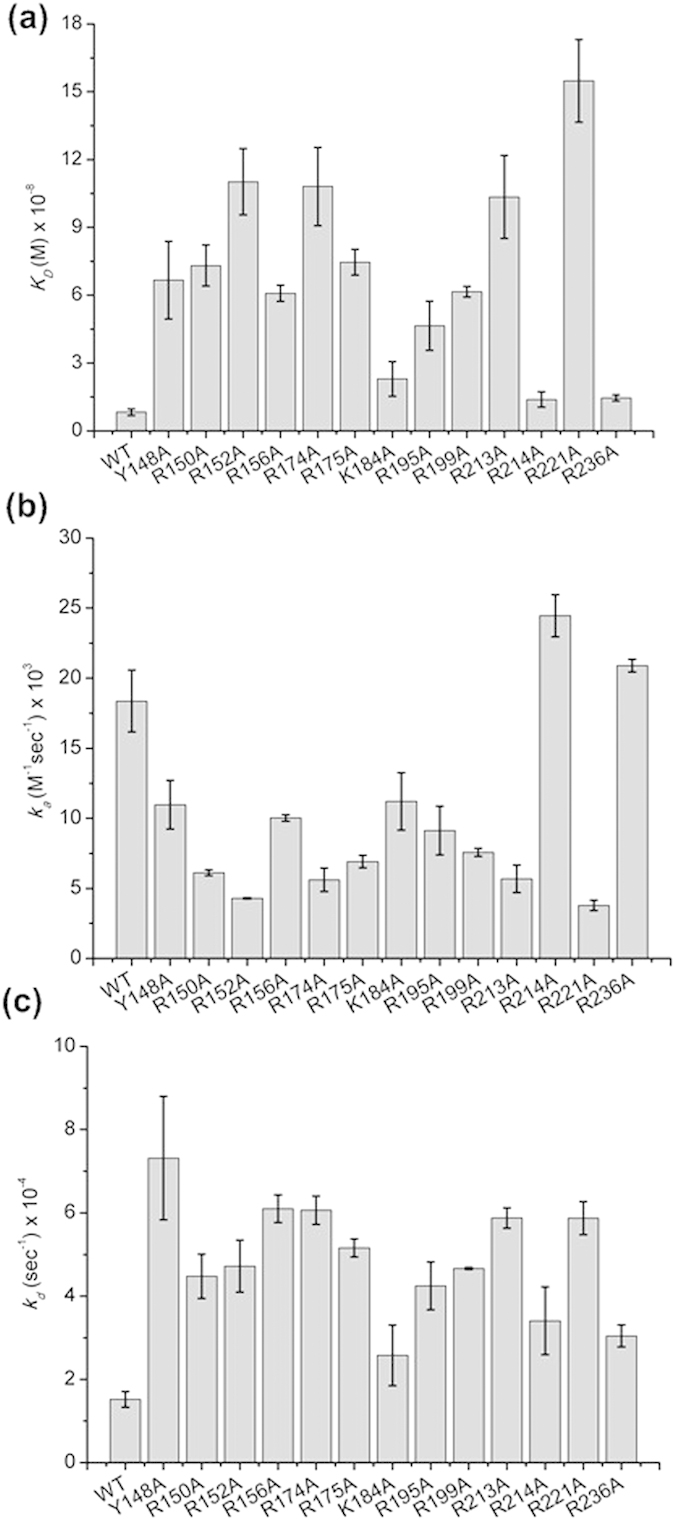
Kinetic analyses of influenza A virus (H1N1) wild-type and mutant nucleoproteins binding to RNA (**a**) *K*_D_, (**b**) *k*_a_, and (**c**) *k*_d_.

The crystal structure of NP revealed that the RNA-binding groove was located between the head and body domains on the exterior surface of the NP oligomers, which is exposed and highly accessible. To examine the effects of conserved positively charged residues and the Y148 residue in the RNA-binding groove of NP, the amino acid residues Y148, R150, R152, R156, R174, R175, K184, R195, R199, R213, R214, R221, and R236 were respectively replaced with alanine (Fig. 1). Each form of NP was expressed and purified. Subsequently, SPR analysis was employed to determine the interactions between the full-length NPs and RNA. The repeated intergenic sequence of influenza virus, 5′-bio(UCCAAAC)_4_-3′, was used as a probe in the SPR experiments. The dissociation constants, *K*_D_ (*k*_d_/*k*_a_), for the various NP and RNA complexes were obtained from kinetic analyses of SPR experiments (Fig. 2). The *k*_a_ values for RNA binding to R214A and R236A were similar to that for WT, whereas Y148A, R150A, R152A, R156A, R174A, R175A, K184A, R195A, R199A, R213A, and R221A had smaller *k*_a_ values than that of WT, suggesting these NP residues are likely important for RNA recognition and interaction. The *k*_d_ values for RNA binding to all mutants were larger than that of WT, and the Y148A mutant had the largest *k*_d_ value (5 fold that of WT), indicating the side chain of Y148 increase RNA residence times in NP (Table 1). In addition to K184A, R214A, and R236A, the dissociation constants for RNA binding to Y148A, R150A, R152A, R156A, R174A, R175A, R195A, R199A, R213A, and R221A ranged from 4.5 × 10^–8 ^M to 15 × 10^–8 ^M and were much larger than those for WT (Fig. 2A), implying that these residues contribute more to RNA binding. Previously, Li *et al.* reported that influenza A virus with NP mutations in Y148, R150, R152, R156, R174, R175, R195, R199, R213, and R221 were significantly attenuated^16^. These biological properties of mutant viruses corroborate our observations at the biochemical level.

**Figure 3:**
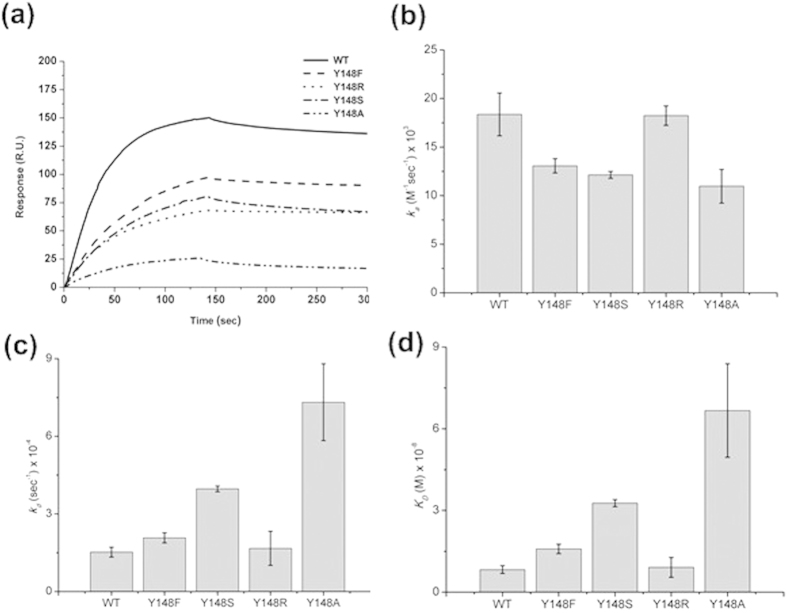
(**a**) The surface plasmon resonance traces form RNA binding affinity assay assessing wild-type and four mutant nucleoproteins. Kinetic analyses of influenza A virus (H1N1) wild-type and mutant nucleoproteins binding to RNA (**b**) *k*_a_, (**c**) *k*_d_, and (**d**) *K*_D_.

One conserved amino acid, Y148, at the end of the RNA-binding groove of the NP was identified as an indispensable residue affecting the RNA binding^18^. To explore the role of Y148 in the interactions between H1N1-NP and RNA, the Y148 residue in the full-length NP was replaced by amino acids with various characteristics (e.g., F, R, S, and A) via site-directed mutagenesis. SPR analyses were then performed to measure the binding affinities between RNA and the purified H1N1-NP (WT and five mutants). Traces from the SPR experiments show the binding capacity of RNA to the WT and mutant NPs. Comparison of the binding capacities of these proteins (Fig. 3A) showed that the binding capacity was decreased in the following order: WT > Y148F > Y148S ~ Y148R > Y148A. These results emphasize the importance of the involvement of Y148 in stacking interactions between the NP and RNA.

The kinetic association and dissociation constants were obtained from analyses of the SPR sensorgrams and the results are listed in Table 1. As shown in Fig. 3B, the *k*_a_ values followed the order WT > Y148R > Y148F ~ Y148S > Y148A, with the *k*_a_ of Y148A smaller than that of the WT by 40.4%. On the other hand, the *k*_d_ values increased in the following order: Y148A > Y148S Y148F > Y148R > WT (Fig. 3C) with the *k*_d_ of Y148A being 4.8 fold larger than that of WT. Taken together, the *K*_D_ values for the H1N1 WT and five mutant NPs decreased in the following order: Y148A > Y148S > Y148F > Y148R > WT (Fig. 3D), implying that the positive charge of Y148R was also involved in the interaction between the NP and RNA.

### Circular dichroism spectral analysis of the thermal stability of WT and mutant nucleoproteins

The conformation of H1N1 WT and mutant NPs including Y148F, Y148S, Y148R, and Y148A with and without RNA were monitored using CD spectroscopy. As shown in Figure S2, the CD spectra of these proteins were scanned from 190 to 250 nm at 25 °C. The CD spectra of H1N1 WT and mutant NPs showed similar well-structured domains with α-helical and β-sheet secondary structures as well as two negative peaks at approximately 205 and 220 nm. Upon addition of RNA, the CD spectra of H1N1 WT and mutant NPs also showed similar intensities at approximately 205 and 220 nm, suggesting that they possessed a similar secondary structure composition upon RNA binding, as compared to H1N1 WT and mutant NPs without RNA.

**Figure 4:**
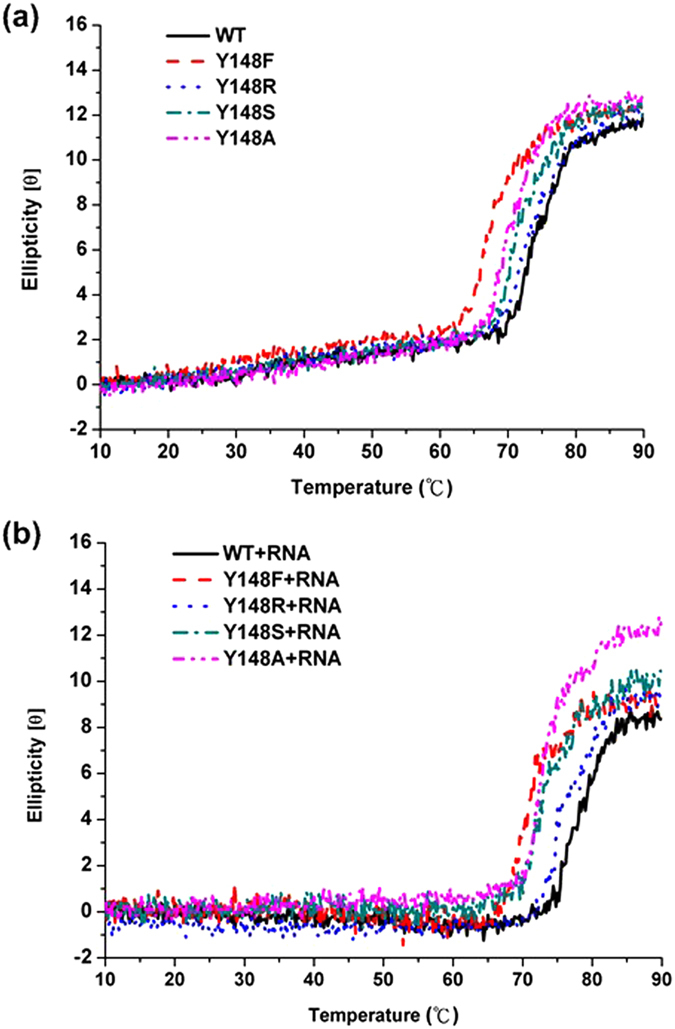
The thermostability measurements of H1N1 WT and mutant NPs (**a**) native proteins, (**b**) native proteins with RNA-binding monitored by circular dichroism at 220 nm. The protein concentration was 10 μM, and the buffer consisted of 50 mM Tris-HCl (pH 7.5) and 150 mM NaCl.

To examine the thermodynamic stabilities of H1N1 WT and mutant NPs including Y148F, Y148S, Y148R, and Y148A, we measured the melting temperatures (*T*_m_) of the purified WT and mutant NPs using CD in which the absorbance at 220 nm was analyzed as a function of temperature (Fig. 4A,B). The WT NP showed a thermal denaturation midpoint at 73.5 °C as determined by CD (Table 2). However, as temperature increases, the mutant NPs unfold more rapidly than the WT NP does, as evidenced by a lower *T*_m_ of approximately 0.7 to 6.2 °C (from 67.3–72.8 °C), indicating that Y148 contributes significantly to the structural stability of the NP. Compared to WT and other mutant NPs, Y148F had a lowest *T*_m_ value. The results indicate that deletion of the tyrosine hydroxyl would significantly reduce the stability of NP, suggesting tyrosine hydroxyl is required for proper NP folding.

We also characterized the stabilizing and structural effects of NP bound to RNA by CD melting analysis (Fig. 4B). The WT NP-RNA complex showed a higher *T*_m_ value (78 °C) than other mutant NP-RNA complexes (Table 2). The *T*_m_ values of the WT and Y148F NPs increased by 3.9 and 3.8 °C, respectively, upon the addition of RNA (Table 2) suggesting that a benzyl side chain has an important effect on the stability of the NP-RNA complex. However, the difference was reduced to 2.5, 1.2 and 2.2 °C for Y148R, Y148S, and Y148 A, respectively, upon addition of RNA. According to previous studies^19^, WT RNP complexes extracted from influenza virus were melt around 63 °C in replicate experiments, compared to 56  °C for protein heated in isolation. Consistent with the early study, our finding show that RNA binding enhance the stability of NP within 10 °C and our result would be meaningful.

### Molecular docking studies to identify compounds that target Y148 of H1N1 nucleoprotein

**Figure 5:**
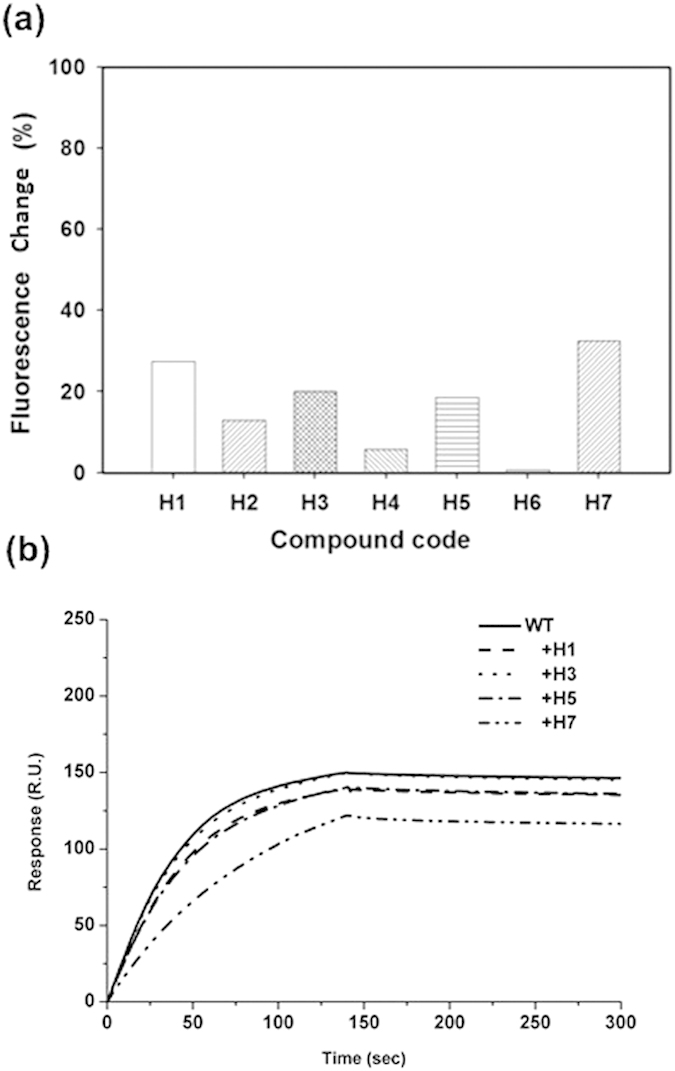
(**a**) Relative fluorescence intensity change of the nucleoprotein of H1N1 (A/HUMAN/TW/2001) strain upon drug (H1–H7) binding at a drug/protein molar ratio of 10. (**b**) sensorgram of the interaction between the immobilized single-stranded RNA and influenza A virus (H1N1) wild-type nucleoproteins in the presence of compounds at 20 μM.

Previous studies have reported that the tyrosine residue in nucleocapsid protein is an attractive target for novel antiviral drug development^20^,^21^. Thus, a virtual screening was performed to target the Y148 of NP. To identify compounds that might interact with the RNA-binding site of NP at Y148, we used the LIBDOCK docking program to evaluate the candidate ligands binding to NP. In the current study, the potential hits with docking scores over 90 were analyzed based on their docking results. We selected seven candidate ligands with high scores (H1 to H7) for the functional assay based on the energy calculated by molecular docking (Table S1). They all contain an aromatic core to stack onto Y148 of the NP. In addition, the aromatic core contains hydrogen bond–forming moieties to mediate the specific interactions with the NP. More importantly, among the 40 potential hits, these seven compounds were readily available. Because our result reported that Y148 is important for NP stability and folding, the top hits were assayed in an *in vitro* drug-induced fluorescence change experiment. The purified recombinant NP was treated with top hits from the in-house compound collection, then the change of tryptophan fluorescence caused by the compound was used to reflect the NP-drug interaction^22^. The four compounds that significantly decreased the NP fluorescence by more than 10% were selected for further characterization (Fig. 5A).

**Figure 6:**
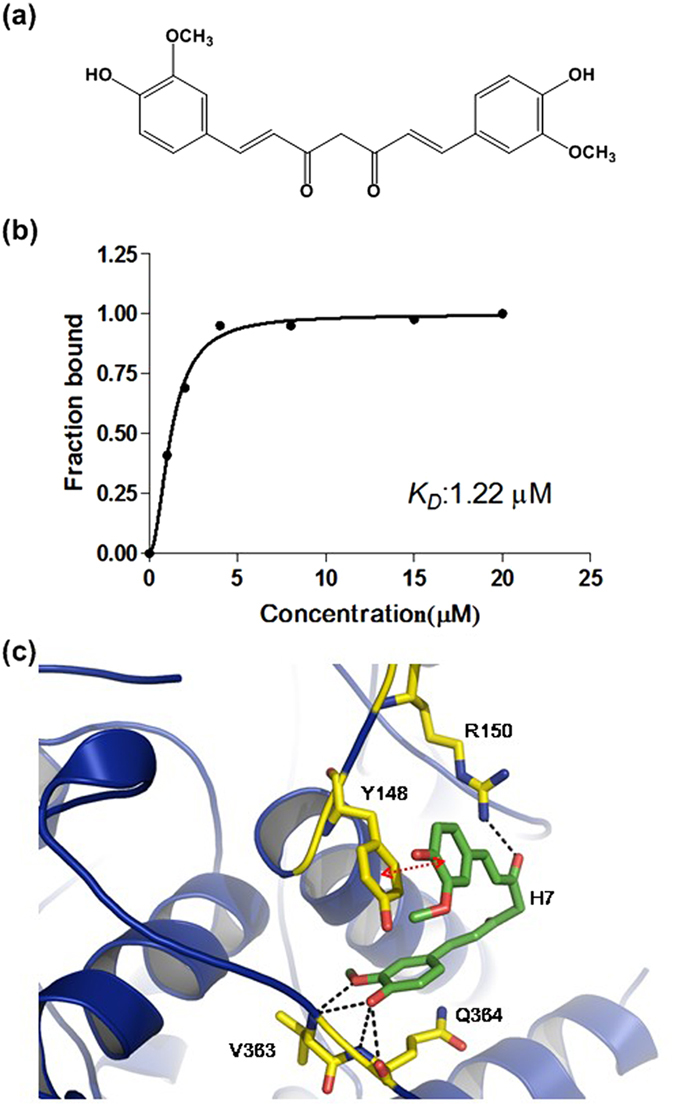
(**a**) Chemical structure of compound H7 [(E,E) -1,7-bis(4-hydroxy-3-methoxyphenyl) -1,6- heptadiene-3,5-dione]. (**b**) Titration of the N protein of influenza virus H1N1 (A/HUMAN/TW/2001) with compound H7. The average of three experiments is shown. The data are expressed as a percentage of the maximal fluorescence change as determined by a fit to the Hill equation. (**c**) The binding model of compound H7 to wild-type nucleoprotein of influenza virus H1N1 (A/HUMAN/TW/2001).

NP possesses multiple RNA binding sites. Compound that targets Y148 in the local area can’t inhibit the binding affinity between RNA and NP absolutely. Due to this reason, we determined the binding affinity in the presence of compounds under saturation conditions at protein: compound ratio of 1:20. We further studied the effects of the four compounds (H1, H3, H5, and H7) on the RNA-binding capacity of NP by SPR experiments. Only H7 decreased the RNA-binding capacity of NP by more than 10% (Fig. 5B). We also evaluated the kinetic association (*k*_a_) and dissociation constants (*k*_d_), respectively, and the dissociation rate constant (*K*_D_) to determine the effects of these four compounds on the RNA-binding affinity toward NP. In the presence of compounds under saturation conditions, the affinity of NP bound to RNA (Fig. 6A) for H7 (*K*_D_ = 19.3 nM) was higher than those for the other compounds. The NP exhibited weaker RNA binding in the presence of H7, with a 2-fold increase in the dissociation constant. The *K*_D_ values for H1, H3, and H5 were 10.4, 9.7, and 11.7 nM, respectively, which are at the same level as the *K*_D_ without drug (Table 3). Figure 5A shows that the NP fluorescence decreased with increasing H7 concentration, suggesting that this decrease reflected the interaction of NP molecules with H7; this result was derived by a Hill plot analysis. Assuming that the amount of quenched fluorescence corresponds to the fraction of NP bound to H7, fitting of the binding curve (Fig. 6B) resulted in an unambiguous 1:1 stoichiometry for the interaction between H7 and NP with a *K*_d_ of 1.22 × 10^–6 ^M. Based on the docking results of NP bound to H7, the methoxyphenyl on the H7 participates in stacking interactions with the Tyr148 side chains. Hydrogen bonds were also formed between R150 and H7 (Fig. 6C). We have analyzed the proposed interactions using in Y148 and R150 mutants. However, we observed that there is relatively low FL change in Y148 and R150 mutants upon addition of H7 by fluorescence titration assay, compared to the wild type, suggesting that H7 inhibits the RNA-binding activity of NP by directly interacting with Y148 and R150 (Figure S3).

### H7 inhibits influenza during the early stage of the virus replication cycle

**Figure 7:**
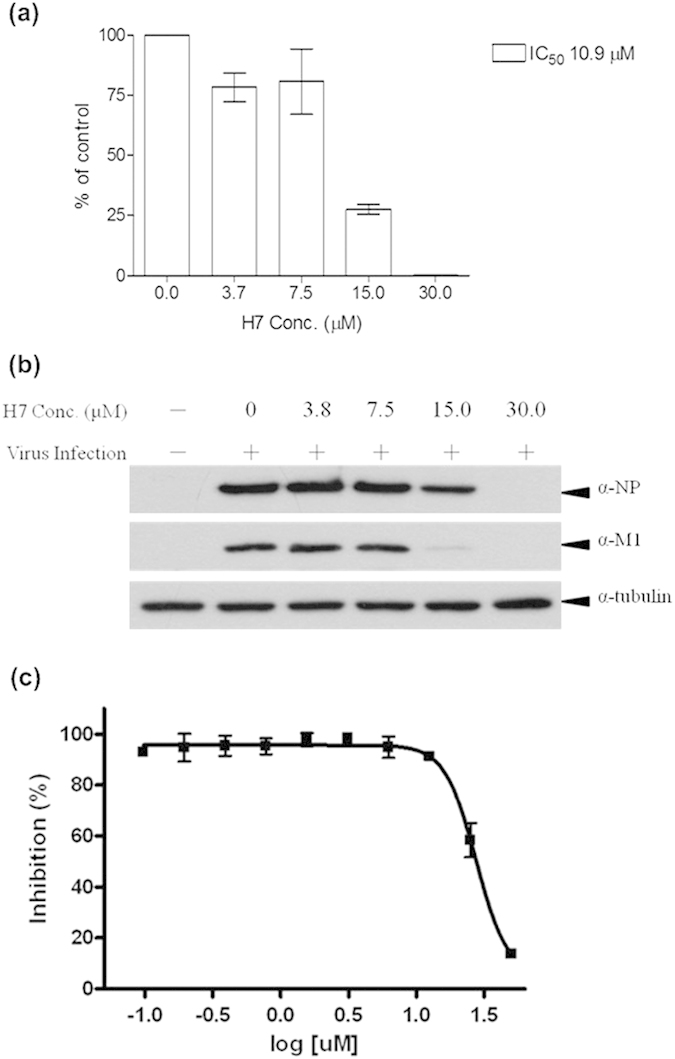
(**a**) The M1 viral RNA synthesis in the influenza virus-infected cells upon H7 treatment. Total cellular RNA was isolated and subjected to RT-PCR and Q-PCR for the detection of H1N1 viral RNA and GAPDH RNA. (**b**) Effects of (E,E)-1,7-bis(4-hydroxy-3-methoxyphenyl)-1,6-heptadiene-3,5-dione (H7) on NP and M1 viral proteins expression. Cellular extracts were isolated and subjected to western blot analysis for the measurement of viral protein α-NP, α-M1 and α -tubulin. A549 cells were mock-infected or infected with H1N1 for 8 h. Various concentrations of H7 were added as indicated at T2 time point. (**c**) The effect of H7 on the cytotoxicity. Cell cytotoxicity of H7 was determined by MTS assay.

To determine if H7 interacts with NP of the influenza A virus, we assessed the effects of H7 after an early step involved in virus replication. NP associates with viral RNA to form a helical nucleocapsid after the first 2 h. We performed a time-of-addition experiment to detect the viral RNA and viral protein synthesis in virus-infected A549 cells. Different concentrations of H7 were added to A549 cells after virus infection (T2: 2 h after infection), and culture supernatants were collected to determine the viral RNA after one viral replication cycle. The matrix protein 1 (M1) viral RNA expression levels during replication were normalized based on the vehicle control (same volume of DMSO added at T2). As shown in Fig. 7A, upon H7 treatment at 15μM, the M1 viral RNA synthesis in the influenza virus–infected cells was reduced by 75% at T2. When the infected cells were treated with 30 μM H7, a similar phenomenon was observed. H7 dose-dependently reduced the viral RNA synthesis during the later steps of the viral cycle after T2. This assay further demonstrated that H7 inhibited influenza virus replication, with an IC_50_ (inhibition concentration at which viral synthesis was reduced by 50%) of 10.9 μM.

We next investigated whether H7 inhibited viral RNA synthesis in virus-infected A549 cells, which would also reduce viral protein synthesis. The expression level of NP in the influenza virus–infected cells as compared to the viral control was reduced by 20% upon H7 treatment in the later steps of the viral cycle. As shown in Fig. 7B, there was a >90% decrease in NP expression. M1 controls the viral RNA levels in the later steps of the viral replication cycle^23^. We also observed the protein expression level of M1 in the virus infected cells at T2 upon H7 treatment. The results suggested that H7 can slow viral protein synthesis after the early stages of viral replication in virus-infected cells. The cytotoxicity of H7 in this study was monitored by MTS assay (Fig. 7C); results indicate that the 50% cytotoxic concentration (CC_50_) value was >27.48 μM. Overall, the time-of-addition experiment indicated that H7 exerts its antiviral effects through RNA-binding capacity with NP after the viral absorption stage.

## Discussion

NP is the most abundant RNA-binding viral protein in influenza virus–infected cells and is responsible for recognizing RNA and forming a filamentous nucleocapsid^24^. It is necessary for viral RNA transcription and replication. Because influenza is a significant threat to both human and avian populations, understanding the molecular mechanisms governing RNP formation may facilitate better control of influenza virus infections^25^. Previous X-ray analyses revealed that folding of the NP is essentially conserved across various influenza virus strains, having a crescent-shaped structure with a head domain, body domain, and tail loop region. The area between the body and head domains is rich in conserved basic residues providing a scaffold for RNA binding, and the tail loop region is involved in the oligomerization of NP^26^.

In this study, we investigated the mechanism of influenza virus NP bound to single-stranded RNA. Using SPR, we explored the effect of single amino acid substitution at 13 hot spots on the surface of the groove between the head and body domains on the RNA-associated properties of NP, including the kinetic behavior of NP bound to RNA, by evaluating the SPR association and dissociation phases. The association phase mainly reflects the entry of RNA into the NP groove, whereas the dissociation phase measures the hydrogen bonding environment within the groove that accommodates the RNA molecules. The structure of the full-length N protein should be taken into consideration in our studies. Moreover, in gel filtration we obtained and checked the uniformity of the purified construct NP. In addition, in the SPR experiment we designed the 28nt in length RNA oligomer, and the length of RNA oligomer can be bound by one protein molecule. During data analysis, we fit the SPR sensorgram of the wild and mutant NP bound to RNA by using 1:1 Langmuir model. We showed that the dissociation rate of NP from the single-stranded RNA increased in all 13 mutants, while the presence of Y148A, R150A, R152A, R156A, R174A, R175A, R195A, R199A, R213A, and R221A mutants significantly decreased the rates of association between NP and RNA, suggesting these 10 residues play a crucial role in RNA binding. Interestingly, the *K*_d_ values of WT, R184A, R214A, and R236A were similar, a finding that is consistent with previous studies showing that the single amino acid substitutions at R184A, R214A, or R236A had no effect on viral-genome replication and transcription^16^. To characterize the interaction of Y148 with RNA, tyrosine was mutated to phenylalanine, serine, and arginine. The association rates (*k*_*a*_) for RNA binding to most of the mutants was decreased significantly, except that the Y148R mutant is similar to that for WT, suggesting that Y148R may participate in RNA-binding of NPs via electrostatic interactions.

**Figure 8:**
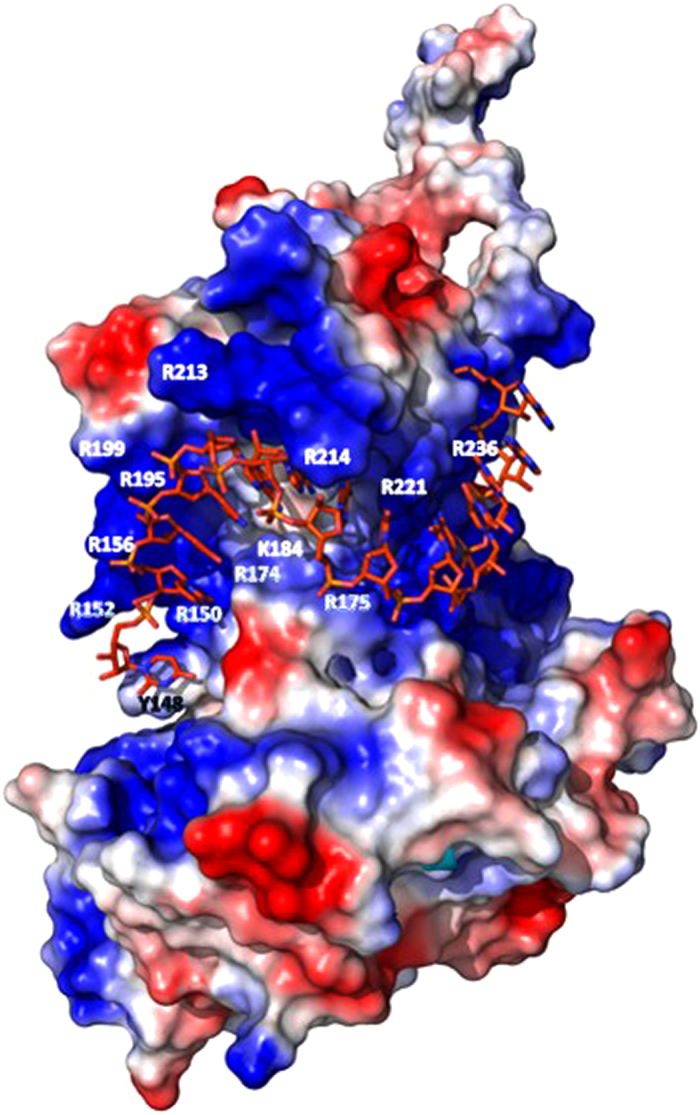
The structural model of wild-type nucleoprotein of influenza virus H1N1 (A/HUMAN/TW/2001) for RNA binding. The amino acids that were mutated in this study are marked.

No structural data were available regarding influenza virus NP binding to single-stranded RNA. Thus, to understand the structural interactions responsible for the RNA recognition by influenza virus NP, we modeled the structure of influenza virus NP in an RNA-bound state (Fig. 8). This model indicates that the RNA-binding groove of the NP contains Y148, R150, R152, R156, R174, R175, K184, R195, R199, R213, R214, R221, and R236, which together clamp the RNA into groove. Y148 stacks against the first nucleotide of the bound RNA to extend the quasi-helix.

Current antiviral drugs developed to treat influenza virus infections primarily target the viral M2 channel and neuraminidase. However, the use of M2 inhibitors, such as amantadine and rimantadine, has been limited by the propensity of these drugs to cause central nervous system side effects and the rapidly increased number of drug-resistant viral strains^27^,^28^. The neuraminidase inhibitors (zanamivir and oseltamivir) are commonly employed to treat influenza virus infections with minimal adverse effects^29^. Yet, recent studies have reported that the influenza virus has started to develop resistance to zanamivir and oseltamivir^30^. Therefore, novel antiviral strategies are required to combat the drug-resistant influenza viruses. The influenza virus NP is a multifunctional RNA-binding protein that is associated with genome and antigenome RNA, and it is necessary for viral RNA transcription and replication^31^. Recent studies suggest that NP represents a potential anti-influenza drug target because of its many crucial functions during the viral life cycle. On the other hands, there are no NP homologous proteins in the cell. Antiviral inhibitors targeting NP may not act nonspecifically on proteins in the cell, causing host cell toxicity and severe side effects. Two strategies to inhibit oligomeric NP function have been reported. The first strategy is to impair normal NP function by interfering with monomer–oligomer equilibrium through either enhancement or inhibition of its oligomerization^32^. Kao *et al.* identified a small-molecule compound called nucleozin that can trigger oligomeric NP aggregates and inhibit their nuclear entry^18^. In addition, Su *et al.* identified several nucleozin analogues that can inhibit the replication of the influenza virus in MDCK cells by inducing the severe aggregation of NP molecules and by preventing RNP formation during the production of viral particles^33^. Gerritz *et al.* discovered a series of influenza replication inhibitors that interfere with NP-dependent processes via formation of higher-order NP oligomers^34^. Shen *et al.* reported that some small molecules targeting E339…R416 from virtual screening were shown to disrupt the formation of NP trimers and inhibit replication of WT and nucleozin-resistant strains^35^. The second strategy is to target the RNA-binding site, which contains several conserved residues^21^,^25^. Lejal *et al.* discovered naproxen, which interferes with the RNA-binding activity of the influenza virus NP and inhibits viral titers^25^. Mutation of conserved residues in the RNA-binding groove of the influenza virus NP led to a significant decrease in its RNA-binding affinity and subsequent decrease in viral replication^16^. According to the protein sequence alignment, the 498-aa NP is highly conserved among all strains of influenza viruses. Because the location of the NP ligand-binding site is highly conserved among influenza viruses, the RNA-binding groove of NP would be a valid target for broad-spectrum antiviral drugs through interference with the RNA-binding activity of the NP. Competitive binders of NP may be employed to combat all strains of influenza virus, including H3N2, H5N1, and influenza B.

According to our mutational analyses of the influenza virus NP, the tyrosine residue on NP (Tyr148) appears to interact with RNA bases via stacking and hydrogen-bonding interactions and to play a crucial role in protein stability. Docking results suggest that several hits can bind to Y148 of the NP-NTD’s groove using virtual screening. Using fluorescence titration and an SPR assay of the NPs, we identified one potential natural compound, curcumin (H7), that targets Y148 of influenza virus NP and potently interferes with its RNA-binding activity. We also found that H7 could inhibit influenza virus replication and the efficacy of H7 is relatively diverse. Previous studies demonstrated that curcumin exist no or low cytotoxicity in normal cells^36^. Importantly, the safety, tolerability, and nontoxicity of curcumin at high doses have been well established by human clinical trials. Therefore, H7 is an ideal point in designing a new class of inhibitors to interfere with RNA-binding activity of NP. Here, we formulated two general guidelines for developing influenza virus NP-Y148 targeting agents. First, a polycyclic aromatic core is required to enable π–π stacking with the tyrosine residues in the NP groove. Second, introducing hydrogen bond–forming moieties to the aromatic core would mediate the specific interactions with the NP. Our findings will be useful for the development of new drugs to disrupt the interactions between RNA and influenza viral NPs.

## Methods

### Expression and purification of full-length recombinant nucleoprotein

To generate full-length wild-type (WT) and mutant NPs encoded by the A/TW/12/2001 (H1N1), NP genes were amplified with the polymerase chain reaction (PCR) using various primers. The PCR products were digested with *Nde*I and *Xho*I, and the DNA fragments were cloned into pET21b (Novagen) using T4 ligase (New England Biolabs). DH5α competent cells that were transformed with the resultant plasmid were grown in culture. Protein expression was induced by supplementing the culture media with 1 mM IPTG, followed by incubation at 10 °C for 24 h. After the bacteria were harvested by centrifugation (6000 × *g* for 15 min at 4 °C), the bacterial pellets were lysed with lysis buffer in the presence of RNase (50 mM Tris-buffered solution [pH 7.5], 100 mM NaCl, 15 mM imidazole). The soluble proteins were isolated from the supernatant following centrifugation (13,000 rpm for 30 min at 4 °C) to remove the precipitate. The recombinant NPs carrying an N-terminal 6×His-tag were purified using a Ni-NTA column (Novagen) with an elution gradient that ranged from 15 to 300 mM imidazole. The pure fractions were collected and dialyzed against the buffer that lacked imidazole. Purified NPs (with greater than 95% purity) were analyzed by gel filtration with Superdex 200 (GE Healthcare) and Coomassie blue staining (Figure S1A). The protein concentrations were determined with the Bradford method using Bio-Rad protein assay reagents.

### Surface plasmon resonance binding experiments

The affinity, association, and dissociation between the NPs and RNA were measured using a BIAcore 3000A surface plasmon resonance (SPR) instrument (Pharmacia, Uppsala, Sweden) equipped with a SA5 sensor chip from Pharmacia. The repeated sequence, 5′-bio(UCCAAAC)_4_-3′, was used as a probe in our SPR experiments. Experiments were conducted by injecting NP at different concentrations in 50 mM Tris (pH 7.5) with 150 mM NaCl (Figure S1B). We calculated the stoichiometric ratio (**S**_**m**_) between NP and RNA, based on the equation,


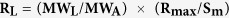


R_L_ [RU] = Immobilization level, R_max_ [RU] = Maximum binding response,

MW_A_ [Da] = Molecular weight of analyte, and MW_L_ [Da] = Molecular weight of ligand.

The S_m_ values of various NPs were calculated to be 0.8 ~ 1.3. Thus, we can ensure that the binding stoichiometries of RNA to various NPs are 1 to 1 and compare the effect of NP mutations on the RNA-binding activity of the protein. The sensorgrams were fit to the 1:1 Langmuir model using the BIAevaluation software (version 3) to determine the association and dissociation rate constants (*k*_a_, *k*_d_).

### Circular dichroism spectroscopy

The circular dichroism (CD) spectra were obtained using a JASCO-815 CD spectropolarimeter. The temperature was controlled by circulating water at the desired temperature in the cell jacket. Each protein was dissolved in 50 mM Tris-HC (pH 7.3) and 150 mM NaCl. The CD spectra were collected between 250 and 190 nm with a 1-nm bandwidth at 1-nm intervals. All of the spectra were obtained from an average of five scans. The photomultiplier absorbance did not exceed 600 V during the analysis. The CD spectra were normalized by subtraction of a background scan with buffer alone or RNA alone. The mean residue ellipticity, [θ], was calculated based on the equation


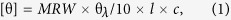


where *MRW* is the mean residue weight, θ_λ_ is the measured ellipticity in millidegrees at wavelength λ, l is the cuvette path length (0.1 cm), and *c* is the protein concentration in g/mL. In addition, the *T*_m_ was determined from the polynomial fitting of the observed curve and taken as the temperature corresponding to half denaturation of the NP. The first derivative of the absorption with respect to temperature, d*A*/d*T*, of the melting curve was computer generated and used to determine the *T*_m_.

### Fluorescence spectroscopy and compounds

The small molecules tested were primarily obtained from an in-house collection of compounds and were included for testing of NP inhibitors. In the drug-induced fluorescence change experiment, a final concentration of 4 μM NP was added to a buffer that contained various concentrations of compound, and the samples were incubated at 25 °C for various durations. The buffer consisted of 50 mM Tris (pH 7.5) and 100 mM NaCl. The tryptophan fluorescence was measured using a Hitachi F-4500 fluorescence spectrophotometer that was equipped with a cuvette with a 1-cm light path. The excitation wavelength was 288 nm, and the emission data were collected between 300 and 400 nm. All static measurements were recorded in triplicate. The relative fluorescence titration intensity was determined using the following equation:


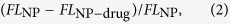


where *FL*_NP_ is the NP fluorescence in the absence of a test compound and *FL*_NP-drug_ is the fluorescence of the NP-compound complex.

To determine the binding constants between a test compound and the various NPs, the compound-induced fluorescence changes (Δ*F*) from three separate experiments at 3 h after addition of a test compound were averaged and fit with the Hill equation using the GraphPad Prism software program (San Diego, CA) as follows:


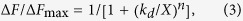


where Δ*F*_max_ is the saturating value of the fluorescence change, *X* is the drug concentration, *k*_d_ is the dissociation constant, and *n* is the Hill coefficient. To exclude the inner filter effects caused by ligand, a correction for the inner filter effect for the binding of H7 and NP is employed by the following equation:^37^


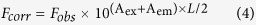


L is the pathlength of the cuvette used, the A terms are the absorbance at the excitation and emission wavelengths, and *F* values are the corrected and observed fluorescence intensities.

### Small-molecule screening against nucleoprotein

The LIBDOCK molecular docking software was used to screen for small molecules that may bind to a structure of the Y148 of NP. The molecules comprised more than 20,000 compounds from several drug databanks in the ZINC database. The binding pocket of the NP, which includes Tyr148 and R150, was represented by a set of spheres during the docking process.

### Cell culture and virus isolation and propagation

Human alveolar basal epithelial cells, A549 cells (ATCC accession no. CCL-185), were grown in RPMI1640 medium (Gibco). Madin-Darby canine kidney (MDCK) cells (ATCC accession no. NBL-2) were grown in Dulbecco’s modified Eagle medium (Gibco). All media were supplemented with 10% fetal bovine serum (Gibco), 100 U/mL of penicillin and streptomycin, and 2 mM L-glutamine. WSN viruses were provided by the Clinical Virology Laboratory of Chang Gung Memorial Hospital (Linkou, Taiwan). Viruses were amplified by using MDCK cells. Virus titer was determined by a plaque assay using MDCK cells.

### Time-of-addition experiment

In the time-of-addition experiment, A549 cells were infected with WSN (MOI 1) and incubated in E0 medium; at T2 time points after infection, H7 was added at different concentrations. The cells were rinsed with PBS to remove the drug and culture medium, and the cell lysates were applied to detect the viral RNA and viral protein at 8 h post-infection.

### Detection of viral RNA in virus-infected cells

A total of 5 × 10^5^ A549 cells were seeded into six-well plates, allowed to reach confluence, and then challenged with virus (MOI 1). Total RNA was extracted from cells using the TRIzol reagent (Invitrogen, Carlsbad, CA, USA). Following phenol-chloroform extraction and isopropanol precipitation, the RNA pellet was washed, dried, and dissolved in 20 mL of RNase-free water. The protocol of RT-PCR amplifications and Q-PCR were as described by Hsu *et al*^38^.

### Western blotting

The cell lysates were collected and lysed with lysis buffer (150 mM NaCl, 1% CA630, 50 mM Tris-base, pH 8.0) for detecting the target proteins. Proteins with sample buffer were subjected to SDS-PAGE and electroblotted onto Hybond ECL membranes (Amersham). Blocking and incubation with antibodies were done using 0.05% Tween 20 and in Tris-buffered. Western blot membranes were incubated with 1:10000 dilution of anti-NP rabbit polyclonal Ab (kindly provided by Dr. Shin Ru Shih, Research Center for Emerging Viral Infectious, Chang Gung University).

### Cytotoxicity Assay

Cell cytotoxicity of inhibitors was determined by MTS assay. A549 cells were grown (5000 cells/well) in 96-well plate for 24 h. The medium was replaced with that containing serial diluted compound after virus infection (T2: 2 h after infection) and the cells were further incubated for 8 h. The culture medium were removed and added 100 μL including MTS and PMS mixture solution, the plate was incubated for 30 min. MTS and PMS are purchased from sigma and prepared in PBS (phosphate buffered saline). To identify a 96-well microtiter plate, 2 ml reagent containing both MTS and PMS at the ratio of 20:1 was mixed immediately with 8ml serum-free DMEM. Each drug concentration was performed with four repeats. The optical density was measured at OD490nm in multi-well spectrophotometer (ELISA reader).

### Molecular modeling

The crystal structure of the influenza A virus NP (Protein Data Bank code 2IQH) lacks a defined tertiary structure in the regions between amino acid residues 73–91, 397–401, and 429–437. The SWISS-MODEL program was used to model the complete structure of the NP. We used the complete structure of the NP as a template to construct a plausible NP-ssRNA complex using the molecular modelling programs Discovery Studio 2.5 and HADDOCK^39^. The RNA force field parameters of Parkinson *et al.* were used^40^. The quality of the model geometry was evaluated using the RMS derivation of the bond length and bond angle. The quality of the modeled structure was tested with PROCHECK^41^

## Additional Information

**How to cite this article**: Liu, C.-L. *et al.* Using mutagenesis to explore conserved residues in the RNA-binding groove of influenza A virus nucleoprotein for antiviral drug development. *Sci. Rep.* **6**, 21662; doi: 10.1038/srep21662 (2016).

## Supplementary Material

Supplementary Information

## Figures and Tables

**Table 1:** The RNA binding kinetic analysis of H1N1 wild-type and mutant nucleocapsid proteins.

Sample	*k*_a_ (M^−1^ s^−1 ^× 10^4^)	*k*_*d*_(s^−1 ^× 10^−4^)	*K*_*D*_(nM)
WT	1.83 ± 0.22	1.52 ± 0.19	8.27 ± 1.43
Y148F	1.30 ± 0.07	2.07 ± 0.19	15.87 ± 1.74
Y148S	1.21 ± 0.04	3.97 ± 0.11	32.69 ± 1.30
Y148R	1.82 ± 0.10	1.67 ± 0.66	9.14 ± 3.65
Y148A	1.09 ± 0.17	7.31 ± 1.49	66.71 ± 17.19

**Table 2:** The thermostability of H1N1 wild-type and mutant nucleocapsid proteins.

Sample	NP *T*_m_ (°C)	NP + RNA *T*_m_ (°C)	Δ*T*_m_ (°C)
WT	73.5	77.4	3.9
Y148F	67.3	71.1	3.8
Y148R	72.8	75.3	2.5
Y148S	71.2	72.4	1.2
Y148A	70.1	72.3	2.2

**Table 3:** The RNA binding kinetic analysis of H1N1 wild-type nucleocapsid protein in the presence of H1, H3, H5, or H7 compounds.

Sample	*k*_d_(S^–1 ^× 10^–4^)	*k*_a_(M^–1 ^S^–1 ^× 10^4^)	*K*_D_(nM)
WT	1.52 ± 0.19	1.83 ± 0.22	8.27 ± 1.43
H1	1.73 ± 0.24	1.66 ± 0.32	10.42 ± 2.48
H3	1.72 ± 0.41	1.78 ± 0.14	9.66 ± 2.43
H5	1.86 ± 0.31	1.59 ± 0.17	11.69 ± 2.32
H7	1.74 ± 0.18	0.90 ± 0.09	19.33 ± 2.78

H1: 3,3′-Methylenebis(4-hydroxycoumarin); H3: N-2-,N-2-Dimethyl-N-1-(6-oxo-5,6-dihydrophenanthridin-2-yl)glycinamide; H5: N,N-Bis(2,5-dihydroxybenzylidene)ethylenediamine; H7: (E,E) -1,7-bis(4-hydroxy-3-methoxyphenyl)-1,6-heptadiene-3,5-dione.
