# Supplementary material for: Using mutagenesis to explore conserved residues in the RNA-binding groove of influenza A virus nucleoprotein for antiviral drug development
Source: Sci Rep. 2016 Feb 26;6:21662. doi: 10.1038/srep21662 (PMC4768256; doi:10.1038/srep21662)
Supplement: Supplementary Information [file srep21662-s1.doc]

**Supplementary Information**

**Using mutagenesis to explore conserved residues in the RNA-binding groove of influenza A virus nucleoprotein for antiviral drug development**

**Chia-Lin Liu^1†^, Hui-Chen Hung ^2†^, Shou-Chen Lo^3^, Ching-Hui Chiang^1^, I-Jung Chen^2^, John T.-A. Hsu^2,*^ and Ming-Hon Hou^1,3,4,*^**

^1^ National Chung Hsing University, Department of Life Science, Taichung, 40227, Taiwan

**^2^** National Health Research Institutes, Institute of Biotechnology and Pharmaceutical Research, Miaoli, 35053, Taiwan

^3^ National Chung Hsing University, Institute of Genomics and Bioinformatics, Taichung, 40227, Taiwan

^4^ National Chung Hsing University, Institute of Biotechnology, Taichung, 40227, Taiwan

*corresponding to [mhho@dragon.nchu.edu.tw](mailto:mhho@dragon.nchu.edu.tw), and [tsuanhsu@nhri.edu.tw](mailto:tsuanhsu@nhri.edu.tw)

† These authors contributed equally to this work

**Table S1.** The chemical structures of H1, H2, H3, H4, H5, H6, and H7 compounds.

| **Number** | **H1** |
| --- | --- |
| **Compound** | **3,3'-Methylenebis(4-hydroxycoumarin)** |
| **Chemical structure** |  |
| **Number** | **H2** |
| **Compound** | **6-chloro-7-(2-morpholin-4-yl-ethylamino)quinoline-5,8dione** |
| **Chemical structure** |  |
| **Number** | **H3** |
| **Compound** | **N-2-,N-2-Dimethyl-N-1-(6-oxo-5,6-dihydrophenanthridin-2-yl)glycinamide** |
| **Chemical structure** |  |
| **Number** | **H4** |
| **Compound** | **7-Allyl-7,8-dihydro-8-oxoguanosine** |
| **Chemical structure** |  |
| **Number** | **H5** |
| **Compound** | **N,N-Bis(2,5-dihydroxybenzylidene)ethylenediamine** |
| **Chemical structure** |  |
| **Number** | **H6** |
| **Compound** | **N-Benzoyl-Gly-His-Leu, N-Hippuryl-L-histidyl-L-leucine hydrate** |
| **Chemical structure** |  |
| **Number** | **H7** |
| **Compound** | **(1E,6E)-1,7-bis(4-hydroxy-3-methoxyphenyl)-1,6-heptadiene-3,5-dione** |
| **Chemical structure** |  |

**Figure S1.** A. The SDS-PAGE analysis of wild-type and mutant Nucleoprotein of influenza virus H1N1 (A/HUMAN/TW/2001). B. The SPR traces form RNA binding affinity assay assessing WT NP at various concentrations.

**Figure S2.** The CD spectra of influenza virus A (H1N1) WT and mutant NPs with and without RNA-binding. The protein concentration was 10 μM, and the buffer consisted of 50 mM Tris–HCl (pH 7.5) and 150 mM NaCl.

**Figure S3.** Relative fluorescence intensity change of the nucleoprotein of H1N1 (A/HUMAN/TW/2001) strain upon drug H7 binding at a drug/protein molar ratio of 5.
